# High-Quality Genome Assembly of *Fusarium oxysporum* f. sp. *lini*

**DOI:** 10.3389/fgene.2020.00959

**Published:** 2020-08-27

**Authors:** George S. Krasnov, Elena N. Pushkova, Roman O. Novakovskiy, Ludmila P. Kudryavtseva, Tatiana A. Rozhmina, Ekaterina M. Dvorianinova, Liubov V. Povkhova, Anna V. Kudryavtseva, Alexey A. Dmitriev, Nataliya V. Melnikova

**Affiliations:** ^1^Engelhardt Institute of Molecular Biology, Russian Academy of Sciences, Moscow, Russia; ^2^Federal Research Center for Bast Fiber Crops, Torzhok, Russia; ^3^Moscow Institute of Physics and Technology, Dolgoprudny, Russia

**Keywords:** *Fusarium oxysporum* f. sp. *lini*, *Linum usitatissimum* L., *de novo* genome assembly, Nanopore, Illumina, genome assemblers, assembly polishers, pure high-molecular-weight DNA

## Abstract

In the present work, a highly pathogenic isolate of *Fusarium oxysporum* f. sp. *lini*, which is the most harmful pathogen affecting flax (*Linum usitatissimum* L.), was sequenced for the first time. To achieve a high-quality genome assembly, we used the combination of two sequencing platforms – Oxford Nanopore Technologies (MinION system) with long noisy reads and Illumina (HiSeq 2500 instrument) with short accurate reads. Given the quality of DNA is crucial for Nanopore sequencing, we developed the protocol for extraction of pure high-molecular-weight DNA from fungi. Sequencing of DNA extracted using this protocol allowed us to obtain about 85x genome coverage with long (N50 = 29 kb) MinION reads and 30x coverage with 2 × 250 bp HiSeq reads. Several tools were developed for genome assembly; however, they provide different results depending on genome complexity, sequencing data volume, read length and quality. We benchmarked the most requested assemblers (Canu, Flye, Shasta, wtdbg2, and MaSuRCA), Nanopore polishers (Medaka and Racon), and Illumina polishers (Pilon and POLCA) on our sequencing data. The assembly performed with Canu and polished with Medaka and POLCA was considered the most full and accurate. After further elimination of redundant contigs using Purge Haplotigs, we achieved a high-quality genome of *F. oxysporum* f. sp. *lini* with a total length of 59 Mb, N50 of 3.3 Mb, and 99.5% completeness according to BUSCO. We also obtained a complete circular mitochondrial genome with a length of 38.7 kb. The achieved assembly expands studies on *F. oxysporum* and plant-pathogen interaction in flax.

## Introduction

Flax (*Linum usitatissimum* L.) is widely used for the production of seeds and fiber. Flax seeds are rich in healthy alpha-linolenic acid (omega-3), lignans, and soluble dietary fibers. They are of great medicinal and nutraceutical value (Goyal et al., 2014; Kezimana et al., 2018; Cullis, 2019) and are potential functional animal feed for improving reproductive capacity and quality of meat, eggs, and milk (Yi et al., 2014; Dutra et al., 2019; Head et al., 2019; Isenberg et al., 2019; Marino et al., 2019; Mattioli et al., 2019). Linseed oil is used in the production of paints, enamels, and resins (Baroncini et al., 2016; Fombuena et al., 2019), while flax fiber is used in textile and composite industries (Muir and Westcott, 2003; Costa et al., 2018; Fombuena et al., 2019; Hu et al., 2019). Fungal pathogens cause diseases in flax that have the greatest negative impact on yield and quality of products worldwide. *Fusarium oxysporum* f. sp. *lini* is responsible for one of the most widespread diseases of flax – fusarium wilt, which leads to estimated yield losses of 20% and in some cases up to 100% (Nyvall, 1989). The pathogen is soil-borne, and infection occurs mainly through the roots. Then it spreads inside the vascular tissues, causing water and nutrient blocking, and eventually leading to wilting, yellowing and browning of top parts of the plants, and finally death (Nair and Kommedahl, 1957; Kroes et al., 1998; Michielse and Rep, 2009). Isolates of *F. oxysporum* f. sp. *lini* are varied and can differ in morphology, physiology, and pathogenicity (Kommedahl et al., 1970; Saharan et al., 2005; Loshakova et al., 2014). Molecular genetic studies are the basis for revealing the origins of the diversities in these traits. In the present study, using the combination of Oxford Nanopore Technologies (ONT) and Illumina platforms, a highly pathogenic isolate of *F. oxysporum* f. sp. *lini*, the most harmful pathogen of flax, was sequenced for the first time.

## Materials and Methods

### Materials

Pathogenic isolate #39 of *F. oxysporum* f. sp. *lini* was provided by the Institute for Flax (Torzhok, Russia). The fungal mycelium was grown on potato dextrose agar (Alpha Biosciences, United States) for 3 weeks.

### DNA Extraction and Purification

Using a sterile scalpel, 1 g of the upper layer of agar with germinated mycelia of *F. oxysporum* was collected and triturated in a mortar with liquid nitrogen. Then, 10 ml of a Carlson lysis buffer [100 mM Tris–HCl pH 9.5 (VWR Life Science, United States); 2% CTAB (VWR Life Science); 1.4 M NaCl (Scharlab, Spain); 1% PEG 8000 (PanReac AppliChem, Germany); 20 mM EDTA (Promega, United States)] that was prewarmed to 65°C and supplemented with 20 μl of β-mercaptoethanol (Bio-Rad, United States) and 0.1 g of PVP K30 (PanReac AppliChem) were added. The homogenate was incubated at 65°C for 1 h 30 min, with stirring every 15 min. Next, an equal volume of chloroform (Acros Organics, United States) was added to the homogenate, stirred on a Thermolyne Maxi Mix III Type 65800 shaker (Thermo Fisher Scientific, United States) at 800 rpm for 10 min, followed by centrifugation at 12000 *g* for 10 min at 4°C. The aqueous phase was transferred to a clean tube with the addition of 0.2 volume of 5x CTAB buffer (5% CTAB, 350 mM EDTA) and incubated at 65°C for 10 min. After that, an equal volume of chloroform was introduced, stirred on a shaker for 10 min, and centrifuged at 12000 *g* for 10 min at 4°C. The aqueous phase was transferred to clean tubes with the addition of 0.7 volume of cold isopropanol (VWR Chemicals, United States), stirred 20 times, and incubated at 72°C for 20 min. It was then centrifuged at 13000 *g* for 10 min at 4°C. Next, the alcohol was collected gently without disturbing the precipitate. The DNA pellet was air-dried for 5 min and dissolved in 2 ml of prewarmed 60°C G-buffer from the Blood & Cell Culture DNA Mini Kit (Qiagen, United States) and incubated at 60°C for 10 min.

To the DNA sample in G-buffer, 4 μl of RNase A (100 mg/ml; 7000 units/ml; Qiagen) was added and incubated at 37°C for 30 min. To this, 25 μl of proteinase K (>600 mAU/ml; Qiagen) was introduced and incubated at 50°C for 40 min. Further, DNA was purified according to the Blood & Cell Culture DNA Mini Kit (Qiagen) protocol. To the DNA elution, 0.7 volume of isopropanol was added and stirred until DNA strands appeared, then, the strands were neatly wrapped around a glass rod. The DNA was transferred to a tube containing a DNA dilution buffer (Evrogen, Russia) and incubated at 50°C for 60 min. The DNA quality and concentration were evaluated on a NanoDrop 2000C spectrophotometer (Thermo Fisher Scientific) and a Qubit 2.0 fluorometer (Life Technologies, United States). The assessment of DNA length and the control of RNA absence were performed by electrophoresis in a 0.8% agarose gel (Lonza, Switzerland).

### DNA Library Preparation and Sequencing on the Illumina Platform

DNA was fragmented on a S220 ultrasonic homogenizer (Covaris, United States), and 1 μg of fragmented DNA was used to prepare the library with the NEBNext Ultra II DNA Library Prep Kit for Illumina (New England Biolabs, United Kingdom) according to the manufacturer’s protocol with size selection of adaptor-ligated DNA of about 600–800 bp. The quality and concentration of the DNA library were evaluated using a 2100 Bioanalyzer instrument (Agilent Technologies, United States) and a Qubit 2.0 fluorometer (Life Technologies), respectively. The resulting DNA library was sequenced on a HiSeq 2500 instrument (Illumina, United States) with a read length of 250 + 250 bp.

### DNA Library Preparation and Sequencing on the ONT Platform

To remove short DNA fragments (up to 10 kb), a Short Read Eliminator Kit (Circulomics, United States) was used. Then, the DNA sample was purified with AMPure XP beads (Beckman Coulter, United States) in a ratio of 1:0.7 (sample:beads).

Preparation of the library was performed using the SQK-LSK109 Ligation Sequencing Kit (ONT, United Kingdom) for 1D genomic DNA sequencing. Minor modifications were introduced to the recommended protocol for library preparation by increasing the incubation time to 20 min at 20°C at the step of DNA recovery and to 60 min at the step of ligation. Sequencing was performed on MinION (ONT) with a FLO-MIN-106 R9.4 flow-cell (ONT).

### Genome Assembly

First, we derived fastq read sequences from MinION raw electric signal fast5 files using guppy 3.2.2 with the high accuracy flip-flop algorithm (dna_r9.4.1_450bps_hac.cfg configuration file). Adapters were trimmed out with Porechop (https://github.com/rrwick/Porechop). Low quality reads (with average Q < 6) were filtered out using trimmomatic 0.38 (Bolger et al., 2014). Illumina reads were trimmed (trailing Q at least 28) and filtered (length < 50) with trimmomatic 0.38.

We used five different assemblers to perform the initial genome assemblies: Canu 1.8 (Koren et al., 2017), Flye 2.6 (Kolmogorov et al., 2019), Shasta 0.4.0 (Shafin et al., 2019), wtdbg2 2.5 (Ruan and Li, 2020), and MaSuRCA 3.3.9 (Zimin et al., 2017). The first four use Nanopore reads and the last one performs a hybrid assembly with Nanopore and Illumina reads. All the parameters were set by default except for the following: minimal read length was set as 3000 bp for Shasta, expected genome size was set as 60 Mb for Flye and wtdbg2. The obtained assemblies were polished using Nanopore reads by Medaka 0.12.1^1^ or Racon 1.4.3 (1–4 iterations) (Vaser et al., 2017). Next, the polished sequences were additionally corrected using Illumina reads by Pilon 1.23 (Walker et al., 2014) or POLCA (built-in MaSuRCA 3.3.9 polisher) (Zimin et al., 2017). Nanopore read mapping to the genome assembly was performed with minimap2 2.17 (Li, 2018), Illumina read mapping – with BWA 0.7.17 (Li, 2013) or bowtie2 2.3.4.1 (Langmead and Salzberg, 2012).

Nx statistics for *F. oxysporum* f. sp. *lini* genome assemblies were calculated using QUAST 5.0.2 (Gurevich et al., 2013). Nx is a specific length for which the subset of contigs of that length or longer covers at least x percent of the assembly (i.e., 50% for N50, 75% for N75, etc.). Using QUAST, we also evaluated the misassembly rates relatively to *F. oxysporum* genomes assembled up to chromosome level and available in the NCBI Genome database.^2^ The completeness of assemblies was evaluated using BUSCO v3 (*Pezizomycotina* odb9 dataset, total 3156 BUSCOs) (Seppey et al., 2019). To visually compare the newly assembled genome of *F. oxysporum* f. sp. *lini* to NCBI chromosome-level assemblies, we created dotplots with the LAST v.1066 aligner (Kielbasa et al., 2011). The percentage of repetitive elements in genome assemblies was identified using RepeatMasker 4.0.9 (rmblast engine).

The best one of the obtained assemblies was chosen for further annotation using the funannotate 1.7.4 pipeline^3^ and previously obtained transcriptome sequencing data (BioProject accession – PRJNA412801). Before annotating, we eliminated redundant contigs using Purge Haplotigs 1.0.4 (Roach et al., 2018).

## Results

The purity of DNA is crucial for Nanopore sequencing, so the protocol was developed to extract DNA of sufficient purity from *Fusarium* fungi. The CTAB-DNA precipitation and Blood & Cell Culture DNA Mini Kit (Qiagen) allowed us to obtain the DNA sample with A260/280 = 1.9 and A260/230 = 2.4. For this sample, DNA concentrations measured with a Qubit fluorometer (Life Technologies) and a Nanodrop spectrophotometer (Thermo Fisher Scientific) had close values that is an important criterion of DNA purity. When DNA was extracted using the CTAB method only, the concentration values differed up to 10-fold and A260/230 values were down to 0.3, indicating significant contamination. The length of Nanopore reads depends on the DNA length and is a key feature affecting the quality of genome assembly. The application of Short Read Eliminator Kit (Circulomics) enabled the removal of short DNA fragments and the average DNA length was about 50 kb. Thus, using the developed protocol of DNA extraction, we obtained long high-purity DNA of *F. oxysporum* f. sp. *lini* that allowed us to receive 312 thousand reads (5.1 Gb) with N50 = 29 kb on MinION (ONT) and 3.5 million 2 × 250 bp reads on HiSeq (Illumina). This corresponds to about 85x coverage with Nanopore reads and about 30x coverage with Illumina reads.

**FIGURE 1:**
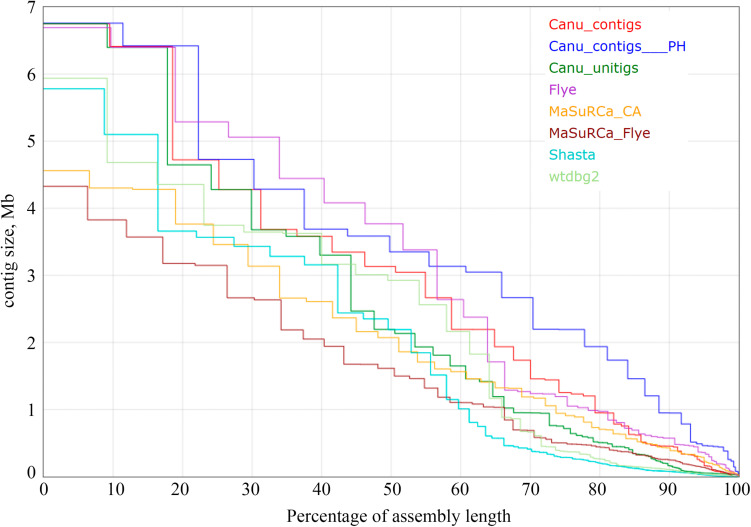
The Nx statistics for *Fusarium oxysporum* f. sp. *lini* assemblies. Results for the following assemblers are presented: Canu 1.8 contigs, Canu 1.8 contigs filtered with Purge Haplotigs (Canu_contigs__PH), Canu 1.8 unitigs, Flye 2.6, MaSuRCA 3.3.9 coupled with Celera Assembler (MaSuRCA_CA), MaSuRCA 3.3.9 coupled with Flye (MaSuRCA_Flye), Shasta 0.4.0, wtdbg2 2.5. Medaka, Racon, Pilon, POLCA polishing did not impact Nx statistics.

**FIGURE 2:**
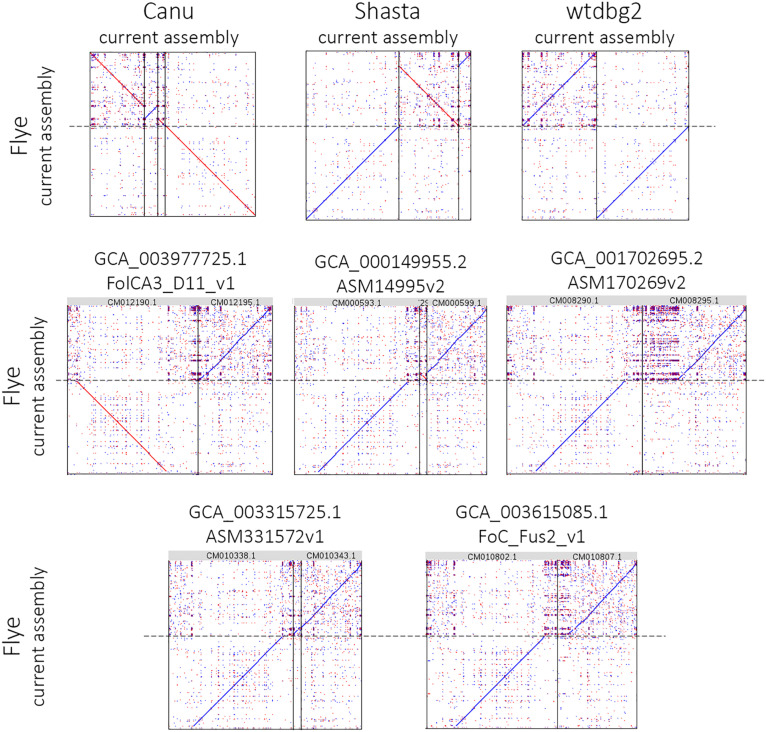
Alignment of the largest Flye 2.6 contig to Canu 1.8, Shasta 0.4.0, wtdbg2 2.5 assemblies and five NCBI chromosome-level assemblies of *Fusarium oxysporum* strains. The chromosome-level assemblies of *F. oxysporum* strains can be found in the NCBI Genome database (https://www.ncbi.nlm.nih.gov/genome/genomes/707).

Using the obtained sequencing data, we benchmarked the five most requested genome assemblers: Canu, Flye, Shasta, wtdbg2, and MaSuRCA. Statistics across all assemblies are represented in Table 1 and Figure 1. Canu produced the longest assembly: 71 Mb (contigs) and 74 Mb (unitigs) with N50 values equal to 3.1 Mb and 2.2 Mb, respectively (according to Canu’s terminology, unitigs are high-confidence contigs). N50 value was the greatest for Flye (3.7 Mb, total length – 69 Mb). However, the first (largest) Flye contig seemed to be misassembled. It represented a combination of several contigs produced by other assemblers – four Canu contigs, three Shasta contigs, or two wtdbg2 contigs. Moreover, this contig did not completely align to any of five NCBI chromosome-level assemblies of *F. oxysporum* strains (Figure 2), and the breakpoint exactly matched to the boundaries of contigs assembled by Canu, Shasta, or wtdbg2. These facts suggested that the first Flye contig was not assembled correctly. One of the possible causes is chimeric Nanopore reads (Xu et al., 2018; Martin and Leggett, 2019). Remarkably, there are repeats upstream of the breakpoint region (Figure 2).

**TABLE 1:** The QUAST statistics across 5 assemblers.

Feature	Canu 1.8 contigs	Canu 1.8 contigs + Purge Haplotigs	Canu 1.8 unitigs	Flye 2.6	MaSuRCa-CA 3.3.9	MaSuRCa-Flye 3.3.9	Shasta 0.4.0	wtdbg2 2.5
Number of contigs (all contigs)	101	35	192	90	87	388	1069	348
Number of contigs (length > 5 kb)	101	35	192	77	87	140	389	345
Number of contigs (length > 50 kb)	75	32	111	55	78	104	137	109
Largest contig, Mb	6.75	6.76	6.75	6.69	4.56	4.33	5.78	5.94
Total length, Mb (all contigs)	70.9	59.2	73.8	69.1	69.2	68.4	66.2	65.0
Total length, Mb (contigs > 5 kb)	70.9	59.2	73.8	69.1	69.2	67.8	65.0	65.0
Total length, Mb (contigs > 50 kb)	70.0	59.1	71.0	68.7	68.9	67.3	61.6	60.8
N50, Mb	3.13	3.35	2.19	3.77	2.07	1.61	2.19	2.93
N75, Mb	1.25	2.19	0.72	1.19	0.94	0.50	0.29	0.37
L50	8	7	9	7	11	13	10	9
L75	17	12	24	16	23	32	35	24
GC, %	47.95	47.98	47.91	47.99	48.13	48.08	48.03	47.99

*Canu contigs + Purge Haplotigs, contigs derived with Canu and then filtered with Purge Haplotigs; MaSuRCa-CA, MaSuRCa pipeline coupled with Celera Assembler; MaSuRCa-Flye, MaSuRCa pipeline coupled with Flye.*

Using QUAST, we evaluated the consistency between our assemblies and five NCBI chromosome-level assemblies of *F. oxysporum* strains (Supplementary Data S1). It should be noted that we can only compare the number of misassemblies between different assemblers within the same reference genome. Judging by the minimum number of misassemblies, the closest to our genome was the assembly of strain GCA_001702695.2/ASM170269v2 (*F. oxysporum* f. sp. *radicis-cucumerinum*). Comparing our assemblies with the reference ones, it was difficult to give preference to any assembler: different tools led in different parameters. According to one of the key parameters, NA50 (analog of N50 for fragments successfully mapped to reference), Shasta was the most accurate. However, Shasta gave an increased number of contigs and worse statistics without reference.

Next, to improve the contig accuracy, raw assemblies were polished with Nanopore reads, Illumina reads, or a combination of these approaches. To polish contigs with Nanopore reads, we applied Medaka or Racon (1–4 iterations). To evaluate the efficacy of polishing, we assessed contig accuracy by matching them to high-quality Illumina reads (statistics were calculated using FreeBayes/POLCA) or by the presence of highly conservative ORFs characteristic for all fungi (by BUSCO). Racon (2 iterations) slightly outperformed Medaka in the number of point substitutions (Supplementary Data S2). However, Medaka won several times by the number of indels – about 40 thousand after Racon and about 10–15 thousand after Medaka. We saw this reflected in the BUSCO metrics, where Medaka greatly outperformed Racon – 98.4% versus 93.4% (Table 2). Indeed, the presence of frameshift insertions/deletions inside ORF in most cases leads to the fact that the protein is no longer detected by BUSCO (or considered as “fragmented BUSCO”). Medaka handled homopolymers well, and the contigs polished with it contained much less false indels. We also noticed that the 2nd iteration of Racon made a much smaller contribution compared to the 1st one. The 3rd and 4th iterations did not make any contribution and even refuse a negative impact somewhere (Table 2 and Supplementary Data S3). Among four assemblers (Canu, Flye, Shasta, and wtdbg2), after polishing with Medaka or Racon, the smallest number of both point substitutions and indels was revealed for Canu assemblies (Supplementary Data S2).

**TABLE 2:** Results of BUSCO analysis for various combinations of Nanopore and/or Illumina polishers.

Polisher (ONT reads)	Polisher (Illumina reads)	Rank	Complete, %	Complete	Fragmented	Missing	Single-copy	Duplicated
Medaka	Pilon-bt2 + POLCA	8	99.46	3139	5	11	3094	45
Medaka	Pilon-bt2	8	99.46	3139	5	12	3093	45
Medaka	Pilon-BWA	10	99.43	3138	6	12	3092	45
Medaka	POLCA	2	99.49	3140	5	11	3095	45
Medaka	POLCA X2	2	99.49	3140	5	11	3094	45
Medaka	–	14	98.42	3106	23	27	3061	45
Racon	–	18	93.44	2949	109	97	2912	37
Racon X2	Pilon-bt2 + POLCA	2	99.49	3140	5	11	3095	45
Racon X2	Pilon-bt2	12	99.33	3135	9	11	3091	45
Racon X2	Pilon-BWA	13	99.18	3130	12	14	3087	43
Racon X2	POLCA	2	99.49	3140	5	11	3095	45
Racon X2	POLCA X2	2	99.49	3140	5	11	3095	45
Racon X2	–	16	93.60	2954	107	96	2916	38
Racon X3	–	15	93.63	2955	108	93	2917	38
Racon X4	–	17	93.54	2952	110	94	2915	37
–	POLCA X2	10	99.43	3138	6	11	3094	44
–	–	19	88.53	2794	196	166	2760	34
MaSuRCa-CA hybrid assembly		1	99.56	3142	5	9	3083	59
MaSuRCa-Flye hybrid assembly		2	99.49	3140	6	10	3096	44

*Rank, place in the overall ranking according to the number of complete (single-copy + duplicated) BUSCOs; ONT, Oxford Nanopore Technologies; bt2, bowtie2; X2, X3, X4, number of iterations; MaSuRCA-CA, MaSuRCA coupled with Celera Assembler; MaSuRCA-Flye, MaSuRCA coupled with Flye. The results are averaged across four assemblers: Canu (contigs and unitigs), Flye, Shasta, and wtdbg2.*

After the initial polishing with Medaka or Racon, we performed the secondary polishing with Pilon and POLCA using Illumina reads. This allowed us to improve the result of BUSCO analysis from 98.4% (Medaka) and 93.4% (Racon) to 99.2–99.5%. POLCA performed better than Pilon both in terms of speed (a difference of about 10 times) and BUSCO completeness. Nevertheless, based on this, it cannot be said that POLCA is superior to Pilon. The question remains how correctly these polishers work with haplotypes, paralogs, and repetitive elements.

Pilon requires BAM files containing Illumina reads mapped to the genome to be polished. We compared two read mappers, BWA and bowtie2, on the efficacy of polishing. BWA may produce more mapping errors compared to bowtie2 (Thankaswamy-Kosalai et al., 2017). This difference may be greater or smaller depending on read length, genome complexity, and richness in paralogs or repeats. Indeed, bowtie2 performed slightly better than BWA in terms of BUSCO metrics (Table 2). However, these values were lower than those achieved by POLCA (99.49%), which implements only BWA. Thus, “Medaka + POLCA” (or only Medaka if you wish to preserve haplotypes) seems to be the best polishing scheme. However, MaSuRCa, a hybrid assembler utilizing both Nanopore and Illumina reads, provided the best results, surpassing all other assemblers and polishing schemes (99.56% BUSCO completeness; Table 2). But MaSuRCa did not demonstrate high N50 values (Table 1).

Based on Nx and BUSCO statistics and the presence of possible misassemblies, we decided that Canu assembly polished with Medaka and POLCA is the best for further analysis. The length of this assembly was 71 Mb that seemed to be excessive. For comparison, the length of 329 chromosome- and scaffold-level assemblies of *F. oxysporum* strains deposited in the NCBI Genome database (see text footnote 2) varied from 38 to 64 Mb (median length = 53 Mb) and the length of representative *F. oxysporum* genome GCA_000149955.2/ASM14995v2 is 61 Mb.

At the same time, BUSCO analysis revealed a very low number of duplicated BUSCOs (1.4%) in our assembly, and therefore the increased genome length (+15–30%) cannot be ascribed to the haplotype separation. To find out possible reasons, we tested our *F. oxysporum* sample for bacterial contamination. However, among 13400 bacterial genomes from the NCBI Genome database deposited before May 2017 (total 28 Gb), we did not find any significant matches. The abundance of repetitive elements could abrogate or inflate the *F. oxysporum* f. sp. *lini* assembly, but RepeatMasker identified only 0.7% of repeats, so that was not the case also.

Next, we mapped our assembly to itself and five NCBI chromosome-level assemblies of *F. oxysporum* strains (Supplementary Data S4–S8) and found that the vast part of contigs greatly mapped to the reference sequences, but many other genomic regions (whole contigs or fragments of contigs) had multiple short homologies to themselves and other genomic regions. The presence of such regions was the reason for the increased length of our assembly. To eliminate this redundancy, we used the Purge Haplotigs tool, which analyzes read coverage density distribution and filters out haplotypes. It is designed to work with bimodal distribution: one peak (minor, as a rule) corresponds to genomic regions with separated haplotypes, and one peak (major) corresponds to regions with merged haplotypes. Even though we obtained only unimodal coverage density distribution, we managed to exclude the redundant haplotigs by tweaking Purge Haplotigs options (setting “align_cov” as 55% and “m” to the median coverage value). The number of complete BUSCOs remained unchanged. The length of the assembly had successfully reduced from 71 Mb to 59 Mb, L50 from 8 to 7, the total number of contigs from 101 to 35; N50 was increased from 3.1 Mb to 3.3 Mb. It should be noted that several assembled contigs represented whole chromosomes. We also assembled a complete circular mitochondrial genome with a length of 38.7 kb.

The obtained assembly was annotated using the funannotate pipeline. As a result, a total of 19351 gene models containing 19607 transcripts were predicted. About 11 thousand models came from PASA based on our RNA-Seq reads and mapping of Trinity transcripts to the assembled genome (from PASA). These models were used for training Augustus, GlimmerHMM, and SNAP, which along with GeneMark and CodingQuarry predicted the rest gene models included in the annotation (Supplementary Data S9). Among 19351 gene models, 311 refer to tRNA. 11841 gene models were successfully annotated using the Pfam database, 12423 – eggNOG, 1334 – BUSCO (Supplementary Data S10). Only 1032 genes were assigned with gene names (it’s normal since such annotation is based only on curated data of UniProt/SwissProt).

The assembled genome was deposited at DDBJ/ENA/GenBank under the accession WHMS00000000 (BioProject accession – PRJNA578147).

## Discussion

The *F. oxysporum* species includes a wide variety of strains and exhibits a high genetic and functional diversity. There are no morphological differences among pathogenic and non-pathogenic strains, while pathogenic strains have a narrow specificity to the host plant (Nelson et al., 1981; Steinberg et al., 2016). Understanding the molecular basis of pathogenicity in the genus *Fusarium* largely depends on the degree of knowledge on the source material that is achieved through molecular genetic studies.

Genome sequencing of fungal plant pathogens enables understanding of the mechanisms of pathogenesis and identification of the key effector genes that enhance infection (Moller and Stukenbrock, 2017; Plissonneau et al., 2017). Obtaining high-quality genomes is a crucial task. The first *F. oxysporum* genome assembly (f. sp. *lycopersici* strain) was done by Broad Institute in 2007 using Sanger sequencing with 6x coverage. The genome consists of 15 chromosomes and has a total length of about 62 Mb (Ma et al., 2013). In 2018, a new version of the assembly of the *F. oxysporum* f. sp. *lycopersici* genome was obtained using the Illumina platform with 76x coverage (Ayhan et al., 2018). This assembly is used as a representative (default) genome for *F. oxysporum* in the NCBI Genome database. It is known that genotypes of different strains, even within the same species, can significantly differ in the gene copy number, presence or absence of specific genes, structure of repeating non-coding regions, and single nucleotide polymorphisms (Schatz et al., 2014; Thudi et al., 2016; Bayer et al., 2017). Till now, there were no sequenced genomes of *F. oxysporum* f. sp. *lini*, which is a harmful pathogen of flax. To date, within specific for flax pathogens, the genome sequence was available only for *Melampsora lini*, which causes rust. Although the assembly of the *Melampsora lini* genome is far from chromosome level (21130 scaffolds were obtained with N50 equal to 31 kb), this is a valuable source to study the molecular mechanisms of pathogenesis (Nemri et al., 2014). Obtained by us high-quality genome assembly of *F. oxysporum* f. sp. *lini* consists of 35 contigs with a total length of 59 Mb and has 99.5% completeness according to BUSCO that became possible due to the development of sequencing technologies and approaches for genome assembly.

Revolution in sequencing methods began from Sanger sequencing that enabled obtaining complete genomes, then high-throughput next-generation sequencing (NGS) methods were developed and made genome sequencing faster and cheaper, and finally, third-generation technologies allowed single-molecule and long-read sequencing that is crucial for high-quality genome assemblies (van Dijk et al., 2018; Arboleda and Xian, 2019). NGS platforms, such as Illumina, BGI, and Ion Torrent, are cost-effective and produce short reads (up to 600 bp), while third-generation platforms, such as Pacific Biosciences (PacBio) and ONT, result in read length of tens and even hundreds of thousands of nucleotides. Currently, genome assembly using long reads is at its peak (Kono and Arakawa, 2019; Amarasinghe et al., 2020; Giani et al., 2020). Over the past 5 years, since the widespread of ONT and PacBio sequencing, several genome assembly tools based on long noisy reads were developed: miniasm, HGAP (only PacBio), Canu, Falcon, ABruijn, wtdbg, Unicycler (the last one is mainly applied for prokaryotic genomes). Among them, Canu seems to be the most accurate assembler, as it was previously shown (Jayakumar and Sakakibara, 2019). However, it requires lots of CPU time (for example, the present *F. oxysporum* assembly required about 3000 CPU hours; ∼2 days with 64 CPU cores) and can hardly be used for the large genomes. To solve this issue, several novel assemblers were developed during the last year, for example, Shasta assembly took only 11.3 min (5.6 CPU hours), Flye – 98 min (∼75 CPU hours). In some cases, Shasta and Flye can outperform Canu in terms of the number of misassemblies and speed but not Nx stats (Shafin et al., 2019). Strictly speaking, the choice of genomic assembler may depend on the genome (its size, ploidy and allele differences, complexity, presence of repetitive elements, etc.), and various assemblers will perform differently for different genomes.

Hence, in this study, we tested five assemblers to choose the most suitable for our purpose. In general, Canu assembly may be considered the best. Compared to other ONT assemblers (Flye, Shasta, and wtdbg2), it is known for a relatively long time (since 2016). Along with Nx statistics, the most important parameters for assessing the assembly quality are misassembly rate, resolution of repeats and tandem duplications, which are rather difficult to evaluate without close reference. At the raw assembly stage, it is these indicators that are important. The accuracy of the assembled contig sequences (i.e., presence of substitutions and indels) is entirely corrected at the polishing stage.

While the BUSCO completeness of raw contigs produced by four assemblers varied significantly (some assemblers have their polishing modules), after processing with Medaka or Racon (Nanopore reads), the results of BUSCO analysis were almost equal, and after subsequent polishing with POLCA or Pilon (Illumina reads), the differences completely smoothed out. Polishing with Illumina reads has both pros and cons. This procedure allows one to quickly decrease the number of short mismatches and indels, achieving sequence accuracy higher than that obtained by polishing with Nanopore reads only. Nevertheless, some authors write that short reads used for correction will homogenize repeats, mix up haplotypes and close paralogs. Even though Illumina reads are of very high quality, their length isn’t sufficient for the true alignment to be identified, and reads from other repeat instances (or paralogs) are used for correction, resulting in incorrect edits.^4^

## Conclusion

In the present study, we for the first time sequenced the genome of a highly pathogenic isolate of *F. oxysporum* f. sp. *lini* using the Nanopore system generating long noisy reads and the Illumina one with short accurate reads and obtained a high-quality genome assembly by testing the most requested assemblers and polishing tools and choosing the best ones for received data. Besides, the protocol for extraction of pure high-molecular-weight DNA from fungi was developed. The obtained genome will significantly improve the studies on *F. oxysporum* and flax response to this pathogen.

## Data Availability Statement

The datasets generated for this study can be found in the DDBJ/ENA/GenBank under the accession WHMS00000000 (BioProject accession – PRJNA578147).

## Author Contributions

TR, AD, and NM conceived and designed the work. EP, RN, LK, ED, LP, and NM performed the experiments. GK, AK, AD, and NM analyzed the data. GK, EP, AD, and NM wrote the manuscript. All authors agreed with the final version of the manuscript and all aspects of the work.

## Conflict of Interest

The authors declare that the research was conducted in the absence of any commercial or financial relationships that could be construed as a potential conflict of interest.

## Footnotes

1 https://github.com/nanoporetech/medaka

2 https://www.ncbi.nlm.nih.gov/genome/genomes/707

3 https://funannotate.readthedocs.io/en/latest/

4 https://canu.readthedocs.io/en/latest/faq.html

## Supplementary Material

DATA S1 The consistency between the obtained *F. oxysporum* f. sp. *lini* assemblies and five chromosome-level assemblies of *F. oxysporum* strains available in the NCBI Genome database.

DATA S2 The accuracy of contigs polished by Medaka (1 iteration) or Racon (2 iterations).

DATA S3 BUSCO results for various combinations of assemblers, Nanopore polishers, and/or Illumina polishers.

DATA S4 Dotplot illustrating homology between the current *F. oxysporum* f. sp. *lini* assembly (before filtering redundant contigs with the Purge Haplotigs tool) and GCA_000149955.2/ASM14995v2 deposited in the NCBI Genome database (vertical axis).

DATA S5 Dotplot illustrating homology between the current *F. oxysporum* f. sp. *lini* assembly (before filtering redundant contigs with the Purge Haplotigs tool) and GCA_001702695.2/ASM170269v2 deposited in the NCBI Genome database (vertical axis).

DATA S6 Dotplot illustrating homology between the current *F. oxysporum* f. sp. *lini* assembly (before filtering redundant contigs with the Purge Haplotigs tool) and GCA_003315725.1/ASM331572v1 deposited in the NCBI Genome database (vertical axis).

DATA S7 Dotplot illustrating homology between the current *F. oxysporum* f. sp. *lini* assembly (before filtering redundant contigs with the Purge Haplotigs tool) and GCA_003615085.1/FoC_Fus2_v1 deposited in the NCBI Genome database (vertical axis).

DATA S8 Dotplot illustrating homology between the current *F. oxysporum* f. sp. *lini* assembly (horizontal axis; before filtering redundant contigs with the Purge Haplotigs tool) and GCA_003977725.1/FolCA3_D11_v1 deposited in the NCBI Genome database (vertical axis).

DATA S9 Sources of gene models.

DATA S10 Annotation statistics for gene models.
