# Supplementary material for: High-Quality Genome Assembly of Fusarium oxysporum f. sp. lini
Source: Front Genet. 2020 Aug 27;11:959. doi: 10.3389/fgene.2020.00959 (PMC7481384; doi:10.3389/fgene.2020.00959)
Supplement: DATA S2 — The accuracy of contigs polished by Medaka (1 iteration) or Racon (2 iterations). [file Data_Sheet_2.pdf]

Supplementary Data 2. The accuracy of contigs polished by Medaka (1 iteration) or Racon (2 iterations).

Assembler	ONT polisher	Contig accuracy	Substitution errors	Insertion/Deletion errors
Canu_contigs	Medaka	99.9873	384	8 626
Canu_contigs	Racon_X2	99.9432	510	39 770
Canu_unitigs	Medaka	99.9876	541	8 622
Canu_unitigs	Racon_X2	99.9458	571	39 351
Flye	Medaka	99.9832	1 761	9 857
Flye	Racon_X2	99.9398	1 252	40 330
Shasta	Medaka	99.9726	1 422	16 385
Shasta	Racon_X2	99.9334	1 247	42 021
wtdbg2	Medaka	99.9765	1 783	13 370
wtdbg2	Racon_X2	99.9314	1 325	42 767

Note: The accuracy is evaluated by POLCA based on Illimuna reads. ONT – Oxford Nanopore Technologies. Green – the best results, red – the worst results, yellow – average results.
