# Supplementary material for: High-Quality Genome Assembly of Fusarium oxysporum f. sp. lini
Source: Front Genet. 2020 Aug 27;11:959. doi: 10.3389/fgene.2020.00959 (PMC7481384; doi:10.3389/fgene.2020.00959)
Supplement: DATA S3 — BUSCO results for various combinations of assemblers, Nanopore polishers, and/or Illumina polishers. [file Data_Sheet_3.pdf]

Supplementary Data 3. BUSCO results for various combinations of assemblers, Nanopore polishers, and/or Illumina polishers.

Assembler	Polisher (ONT reads)	Polisher (Illumina reads)	Rank	Comp-lete, %	Comp-lete	Frag-mented	Missing	Single-copy	Dupli-cated
Canu (contigs)	Medaka	Pilon + POLCA	**25**	**99.46**	3139	5	12	3094	45
Canu (unitigs)	Medaka	Pilon + POLCA	**10**	**99.49**	3140	5	11	3095	45
Flye	Medaka	Pilon + POLCA	**25**	**99.46**	3139	6	11	3094	45
Shasta	Medaka	Pilon + POLCA	**10**	**99.49**	3140	5	11	3091	49
wtdbg2	Medaka	Pilon + POLCA	**25**	**99.46**	3139	5	12	3096	43
Canu (contigs)	Medaka	Pilon-bt2	**25**	**99.46**	3139	5	12	3094	45
Canu (unitigs)	Medaka	Pilon-bt2	**25**	**99.46**	3139	5	12	3094	45
Flye	Medaka	Pilon-bt2	**10**	**99.49**	3140	6	10	3095	45
Shasta	Medaka	Pilon-bt2	**39**	**99.43**	3138	6	12	3089	49
wtdbg2	Medaka	Pilon-bt2	**39**	**99.43**	3138	5	13	3095	43
Canu (contigs)	Medaka	Pilon-BWA	**39**	**99.43**	3138	6	12	3093	45
Canu (unitigs)	Medaka	Pilon-BWA	**39**	**99.43**	3138	6	12	3093	45
Flye	Medaka	Pilon-BWA	**39**	**99.43**	3138	7	11	3093	45
Shasta	Medaka	Pilon-BWA	**46**	**99.40**	3137	7	12	3088	49
wtdbg2	Medaka	Pilon-BWA	**39**	**99.43**	3138	5	13	3095	43
Canu (contigs)	Medaka	POLCA	**10**	**99.49**	3140	5	11	3095	45
Canu (unitigs)	Medaka	POLCA	**10**	**99.49**	3140	5	11	3095	45
Flye	Medaka	POLCA	**4**	**99.52**	3141	6	9	3096	45
Shasta	Medaka	POLCA	**10**	**99.49**	3140	5	11	3091	49
wtdbg2	Medaka	POLCA	**25**	**99.46**	3139	5	12	3096	43
Canu (contigs)	Medaka	POLCA X2	**25**	**99.46**	3139	5	12	3094	45
Canu (unitigs)	Medaka	POLCA X2	**25**	**99.46**	3139	5	12	3094	45

Flye	Medaka	POLCA X2	**4**	**99.52**	3141	6	9	3096	45
Shasta	Medaka	POLCA X2	**10**	**99.49**	3140	5	11	3091	49
wtdbg2	Medaka	POLCA X2	**25**	**99.46**	3139	5	12	3096	43
Canu (contigs)	Medaka		**62**	**98.67**	3114	18	24	3069	45
Canu (unitigs)	Medaka		**60**	**98.70**	3115	19	22	3070	45
Flye	Medaka		**60**	**98.70**	3115	21	20	3069	46
Shasta	Medaka		**64**	**97.66**	3082	32	42	3035	47
wtdbg2	Medaka		**63**	**98.42**	3106	24	26	3064	42
Canu (contigs)	Racon		**83**	**93.44**	2949	109	98	2912	37
Canu (unitigs)	Racon		**71**	**93.60**	2954	108	94	2918	36
Flye	Racon		**85**	**93.22**	2942	115	99	2907	35
Shasta	Racon		**69**	**93.63**	2955	104	97	2914	41
wtdbg2	Racon		**84**	**93.38**	2947	110	99	2910	37
Canu (contigs)	Racon X2	Pilon + POLCA	**10**	**99.49**	3140	5	11	3095	45
Canu (unitigs)	Racon X2	Pilon + POLCA	**10**	**99.49**	3140	5	11	3095	45
Flye	Racon X2	Pilon + POLCA	**10**	**99.49**	3140	6	10	3096	44
Shasta	Racon X2	Pilon + POLCA	**4**	**99.52**	3141	5	10	3093	48
wtdbg2	Racon X2	Pilon + POLCA	**25**	**99.46**	3139	5	12	3097	42
Canu (contigs)	Racon X2	Pilon-bt2	**52**	**99.33**	3135	10	11	3090	45
Canu (unitigs)	Racon X2	Pilon-bt2	**52**	**99.33**	3135	10	11	3090	45
Flye	Racon X2	Pilon-bt2	**49**	**99.37**	3136	10	10	3092	44
Shasta	Racon X2	Pilon-bt2	**49**	**99.37**	3136	9	11	3089	47
wtdbg2	Racon X2	Pilon-bt2	**54**	**99.30**	3134	8	14	3092	42
Canu (contigs)	Racon X2	Pilon-BWA	**58**	**99.14**	3129	12	15	3086	43
Canu (unitigs)	Racon X2	Pilon-BWA	**57**	**99.18**	3130	12	14	3086	44
Flye	Racon X2	Pilon-BWA	**55**	**99.24**	3132	12	12	3089	43
Shasta	Racon X2	Pilon-BWA	**56**	**99.21**	3131	12	13	3085	46

wtdbg2	Racon X2	Pilon-BWA	**59**	**99.11**	3128	11	17	3087	41
Canu (contigs)	Racon X2	POLCA	**4**	**99.52**	3141	5	10	3097	44
Canu (unitigs)	Racon X2	POLCA	**25**	**99.46**	3139	5	12	3095	44
Flye	Racon X2	POLCA	**10**	**99.49**	3140	6	10	3096	44
Shasta	Racon X2	POLCA	**4**	**99.52**	3141	5	10	3092	49
wtdbg2	Racon X2	POLCA	**46**	**99.40**	3137	5	14	3095	42
Canu (contigs)	Racon X2	POLCA X2	**10**	**99.49**	3140	5	11	3096	44
Canu (unitigs)	Racon X2	POLCA X2	**25**	**99.46**	3139	5	12	3095	44
Flye	Racon X2	POLCA X2	**1**	**99.56**	3142	6	8	3098	44
Shasta	Racon X2	POLCA X2	**4**	**99.52**	3141	5	10	3092	49
wtdbg2	Racon X2	POLCA X2	**39**	**99.43**	3138	5	13	3095	43
Canu (contigs)	Racon X2		**68**	**93.66**	2956	103	97	2916	40
Canu (unitigs)	Racon X2		**78**	**93.50**	2951	111	94	2912	39
Flye	Racon X2		**71**	**93.60**	2954	113	89	2920	34
Shasta	Racon X2		**71**	**93.60**	2954	100	102	2913	41
wtdbg2	Racon X2		**75**	**93.57**	2953	106	97	2919	34
Canu (contigs)	Racon X3		**71**	**93.60**	2954	105	97	2914	40
Canu (unitigs)	Racon X3		**66**	**93.76**	2959	108	89	2922	37
Flye	Racon X3		**78**	**93.50**	2951	118	87	2916	35
Shasta	Racon X3		**67**	**93.73**	2958	102	96	2915	43
wtdbg2	Racon X3		**78**	**93.50**	2951	109	96	2918	33
Canu (contigs)	Racon X4		**78**	**93.50**	2951	113	92	2915	36
Canu (unitigs)	Racon X4		**82**	**93.47**	2950	110	96	2912	38
Flye	Racon X4		**69**	**93.63**	2955	108	93	2921	34
Shasta	Racon X4		**75**	**93.57**	2953	108	95	2910	43
wtdbg2	Racon X4		**75**	**93.57**	2953	110	93	2918	35
Canu (contigs)		POLCA X2	**10**	**99.49**	3140	5	11	3097	43

Canu (unitigs)		POLCA X2	**25**	**99.46**	3139	5	12	3096	43
Flye		POLCA X2	**10**	**99.49**	3140	6	10	3095	45
MaSuRCa-CA		POLCA X2	**1**	**99.56**	3142	5	9	3083	59
MaSuRCa-Flye		POLCA X2	**25**	**99.46**	3139	6	11	3095	44
Shasta		POLCA X2	**49**	**99.37**	3136	9	11	3090	46
wtdbg2		POLCA X2	**46**	**99.40**	3137	6	13	3094	43
Canu (contigs)			**88**	**88.66**	2798	201	157	2764	34
Canu (unitigs)			**87**	**88.69**	2799	201	156	2765	34
Flye			**65**	**93.92**	2964	98	94	2927	37
MaSuRCa-CA			**1**	**99.56**	3142	5	9	3083	59
MaSuRCa-Flye			**10**	**99.49**	3140	6	10	3096	44
Shasta			**86**	**91.10**	2875	152	129	2839	36
wtdbg2			**89**	**80.26**	2533	327	296	2505	28

Note: Rank – place in the overall ranking according to the number of complete (single-copy + duplicated) BUSCOs; ONT – Oxford Nanopore Technologies; bt2 – bowtie2; X2, X3, X4 – number of iterations; MaSuRCA-CA – MaSuRCA coupled with Celera Assembler; MaSuRCA-Flye – MaSuRCA coupled with Flye. Green – the best results, red – the worst results, yellow – average results.
