# Supplementary material for: High-Quality Genome Assembly of Fusarium oxysporum f. sp. lini
Source: Front Genet. 2020 Aug 27;11:959. doi: 10.3389/fgene.2020.00959 (PMC7481384; doi:10.3389/fgene.2020.00959)
Supplement: DATA S5 — Dotplot illustrating homology between the current F. oxysporum f. sp. lini assembly (before filtering redundant contigs with the Purge Haplotigs tool) and GCA_001702695.2/ASM170269v2 deposited in the NCBI Genome database (vertical axis). [file Data_Sheet_5.pdf]

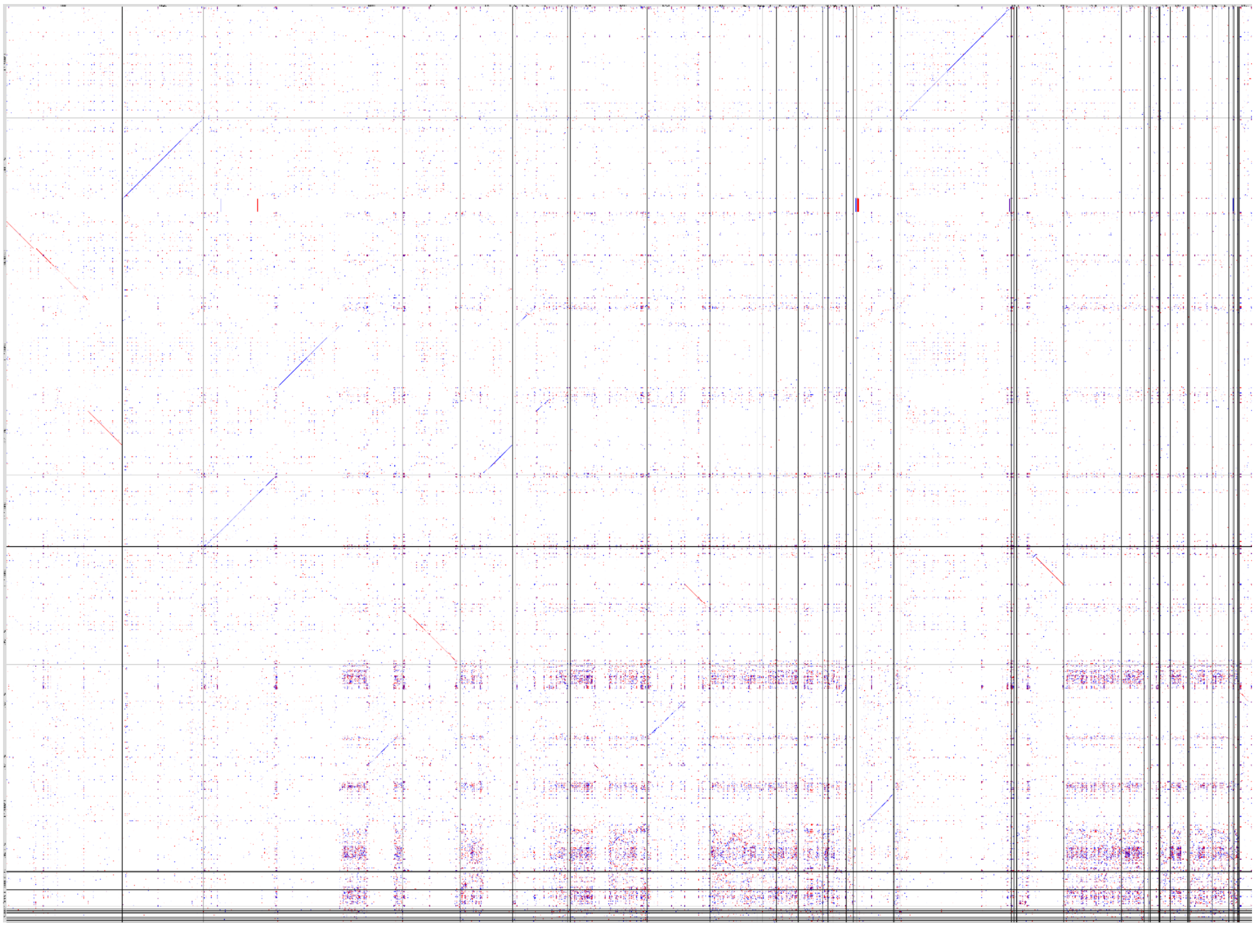

Supplementary Data 5. Dotplot illustrating homology between the current *F. oxysporum* f. sp. *lini* assembly (before filtering redundant contigs with the Purge Haplotigs tool) and GCA_001702695.2 / ASM170269v2 deposited in the NCBI Genome database (vertical axis).
