# Supplementary material for: High-Quality Genome Assembly of Fusarium oxysporum f. sp. lini
Source: Front Genet. 2020 Aug 27;11:959. doi: 10.3389/fgene.2020.00959 (PMC7481384; doi:10.3389/fgene.2020.00959)
Supplement: DATA S9 — Sources of gene models. [file Data_Sheet_9.pdf]

Supplementary Data 9. Sources of gene models.

**Source**	**EVidenceModeler weight**	**Suggested gene models**	**Comments**
Augustus	1	10210	trained on PASA results
Augustus-HiQ	2	6883	trained on PASA results
CodingQuarry	2	21275	uses RNA-Seq mapped reads to derive gene models
GeneMark	1	20931	self-training
GlimmerHMM	1	19009	trained on PASA results
PASA	6	11326	uses mapped Trinity transcripts to derive gene models
SNAP	1	19296	trained on PASA results
Total		108930	
