# Supplementary material for: High-Quality Genome Assembly of Fusarium oxysporum f. sp. lini
Source: Front Genet. 2020 Aug 27;11:959. doi: 10.3389/fgene.2020.00959 (PMC7481384; doi:10.3389/fgene.2020.00959)
Supplement: DATA S10 — Annotation statistics for gene models. [file Data_Sheet_10.pdf]

Supplementary Data 10. Annotation statistics for gene models.

	**Annotated gene models**	**Total annotation entries added**
Pfam	11841	19718
Secretome	1744	1744
MEROPS	539	539
eggNOG	12423	12423
CAZYme	732	1357
BUSCO	1334	1334
antiSMASH	205	266
Assigned gene names	1032	1032
